# Assessing the efficacy of TNF‐alpha inhibitors in preventing emergency and emergent colectomies

**DOI:** 10.1002/jgh3.12229

**Published:** 2019-08-02

**Authors:** Ruben Rajan, Matthew W Trinder, Johnny Lo, Mary Theophilus

**Affiliations:** ^1^ Department of General Surgery Royal Perth Hospital Perth Western Australia Australia; ^2^ School of Science Edith Cowan University Perth Western Australia Australia

**Keywords:** emergency colectomy, inflammatory bowel disease, infliximab, TNF‐∝ inhibitors, ulcerative colitis

## Abstract

**Background and Aim:**

Severe ulcerative colitis (UC) is potentially life threatening and is associated with significant morbidity. TNF‐∝ inhibitors (Infliximab) were introduced in Australia for the management of medically resistant, acute, severe flares of UC in 2008. The aim of this study is to assess the efficacy of Infliximab in preventing emergent and emergency colectomies for patients with moderate to severe UC by comparing colectomy rates before and after its introduction at our institution.

**Methods:**

This was a retrospective cohort study of all patients who were admitted to the Royal Perth Hospital with a flare of UC between 2002 and 2017. Patients were divided into two cohorts: those admitted prior to the introduction of Infliximab (pre‐2008) and those admitted after. We compared data between these two groups, including age, gender, length of admission, use of Infliximab, colectomy, and complications of surgery. We defined emergency surgery as requiring surgery during the index admission and emergent surgery as an operation within 54 weeks.

**Results:**

A total of 313 UC cases from 2002 to 2017 were analyzed. There was a decrease in emergency and emergent colectomies from 19.4 to 8% in the post‐2008 cohort (*P* = 0.008). Furthermore, there was a decrease in the proportion of operations performed as emergencies, from 36 to 20%. This resulted in a significantly reduced length of stay (13.4–9.7 days, *P* < 0.05) and complication rate (36 to 20%, *P* < 0.05).

**Conclusion:**

Overall, the need for emergency and emergent operations has drastically reduced at our institution with the introduction of Infliximab. This study has confirmed the efficacy of Infliximab in reducing colectomy rates at our institution.

## Graphical abstract

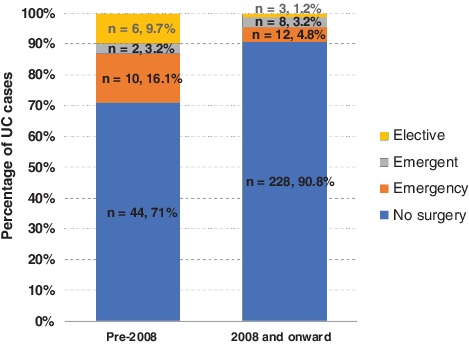

This is a retrospective cohort study that compared the rates of colectomy for severe ulcerative colitis over a 15‐year period before and after the introduction of Infliximab therapy at our institution. We found a significant decrease in the rates of colectomy for severe ulcerative colitis over the study period, as well as a reduction in the rates of complications.

## Introduction

Ulcerative colitis (UC) is a chronic inflammatory disease in the inflammatory bowel disease (IBD) spectrum of the colon and rectum. In Australia, the annual incidence of UC is 17.4 per 100 000, and there are currently 33 000 Australians living with the disease.^1^

The pathogenesis of IBD is thought to be a result of the dysregulation of the mucosal immune system.^2^ Although the exact mechanism by which the inflammatory cascade is stimulated in IBD is unknown, studies have shown that TNF‐∝ mediates inflammation. TNF‐*α* blockade has a restorative function in the suppressive action of regulatory T‐cells, which is the basis of its use in IBD.^3^

The use of TNF‐*α* inhibitors (Infliximab) in the setting of moderate to severe flares of UC was approved by the Therapeutic Guidelines Australia (TGA) in February 2007. Its use is highly regulated given that the cost of a single 100‐mg injection is $507.42. To be eligible, adult patients must first be clinically scored by the Truelove and Witts criteria as having severe disease, have a Mayo Score of >6, or have had a positive documented response previously to TNF‐∝ inhibitors.

Only a few studies have directly compared the incidence of colectomies before and after the introduction of this drug.^4^There are no similar studies in an Australian context. Pooled data from ACT‐1 and ‐2 showed a 7% absolute risk reduction and a hazard ratio of 0.59 for colectomies when comparing TNF‐alpha inhibitor use and placebo.^5^, ^6^

The aim of this study is to assess the efficacy of Infliximab in preventing colectomies in the emergency setting for patients with moderate to severe UC by comparing colectomy rates before and after the introduction of Infliximab at an Australian institution.

## Methods

### *Data collection*

A retrospective cohort study was undertaken of patients who were admitted to the Royal Perth Hospital with an acute flare of ulcerative colitis. The study period was from 1 July 2002 to 1 July 2017. All admissions to the hospital for an acute flare of UC, which were biopsy proven, in patients aged >16 years were included. Patients with Crohn's disease, nonspecific colitis, and all admissions where the primary diagnosis was not consistent with an acute flare of UC were excluded.

Admissions were identified using The Open Web Administration System (TOPAS) and WebPAS (replaced ToPAS from 2012). The International Statistical Classification of Diseases and Related Health Problems 10th Revision, Australian Modification (ICD‐10‐AM) codes: K51.0 Ulcerative (acute) pancolitis; K51.2 Ulcerative (acute) proctitis; K51.3 Ulcerative (chronic) rectosigmoiditis; K51.4 Inflammatory polyps; K51.5 left sided colitis; K51.8 Other UC; and K51.9 UC, unspecified were used to search this database. A total of 3117 admissions were identified. After applying the exclusion criteria, 313 admissions were eligible for analysis.

The variables extracted during chart review included age at admission, gender, length of admission, medication used (corticosteroids, azathioprine/ 6‐MP, Ciclosporin, 5‐ASA), use of TNF‐∝ inhibitors, complications of TNF‐∝ inhibitors, colectomy, complications of surgery, and death. Each admission was also scored according to the Truelove and Witts classification^7^ as this is the accepted criteria for approval of TNF‐∝ inhibitor use in IBD and is easily extractable from medical records. If patients underwent a colectomy, the timing was categorized as Emergency Colectomy or Emergent Colectomy (within 54 weeks of admission). There are currently no internationally accepted definitions to distinguish semiurgent colectomies from true elective colectomies. The 54‐week mark was chosen because the ACT‐1 and ACT‐2 trials demonstrated the maximal efficacy of Infliximab at weeks 30 and 54.^8^ An emergent colectomy was therefore defined as any semielective colectomy performed within 54 weeks of an admission for acute colitis. Admissions that eventually required an elective colectomy at the end of the study period were also recorded for subgroup analysis. Admissions were then divided into two cohorts: pre‐Infliximab use (2007 and before) and postapproval for Infliximab use (2008 onward).

### *Statistical analysis*

Frequency and proportion of colectomies, use of Infliximab, and severity of UC on admission were assessed. Chi‐square tests were used to determine if there was any difference in the proportions of these measures prior to and following the introduction of Infliximab in 2008. Logistic regression was then used to formally examine the relationship between Infliximab and the rate of UC before and after 2008. Odds ratios (ORs) and confidence intervals were presented as model estimates. Data pertaining to hospital length of stay were not normally distributed (Shapiro Wilk *P* < 0.001) and were log‐transformed. A general linear model (GLM) was then used to investigate whether the introduction of Infliximab in 2008 had any impact on patient's hospital length of stay. The age at which UC was presented in patients and the severity of the condition were also adjusted for and included in both the logistic regression model and the GLM. Significance of variables was achieved if *P* < 0.05.

## Results

A total of 313 UC cases from 2002 to 2017 were collated and analyzed. (Table 1). The number of UC admissions pre‐ and post‐Infliximab was 62 and 251, respectively. The median interquartile range (IQR) age at presentation was 35.1 (27.1–47.6) years. One in three admissions was moderate (*n* = 100, 32%), while the remaining were severe (*n* = 213, 68%). There were no admissions for mild flares of UC.

**Table 1:** Patient demographics

Demographics	Pre‐2008 (62)	2008 onwards (251)	*P* value
Age (mean)	39.8	38.5	0.55
Age (SD)	16.5	15.4	0.406
LOS (mean)	10.8	6.2	0.004
LOS (SD)	11.9	5.5	<0.001
Prednisolone	62 (100%)	236 (94%)	0.055
Azathioprine	24 (38.7%)	101 (40.2%)	0.756
5‐ASA	57 (91.9%)	210 (83.7%)	0.175
TNF‐α	4 (6.5%)	155 (61.8%)	<0.001
Ciclosporin	9 (14.5%)	1 (0.4%)	<0.001

There was a gradual increase in the number of admissions involving the use of Infliximab from 2008. In the first 4 years since the introduction of Infliximab, one in four cases (16 of 53, 23%) involved the use of Infliximab, which later increased to one in three (19 of 55, 34.5%) cases in 2012–2013 and then to almost one in two cases (61 of 127, 48%) from 2014 to 2017. Furthermore, 47% (99 of 213) of all severe cases of UC involved infliximab. There was only a single instance where Infliximab was used for moderate UC. This patient had previously presented with a severe flare and had already received Infliximab prior to this admission.

Approximately 13% (*n* = 28) of all severe cases required emergency or emergent colectomies, of which 71% (20 of 28) underwent emergency surgery. Only 4% (*n* = 4) of patients with moderate UC underwent surgery.

**Figure 1:**
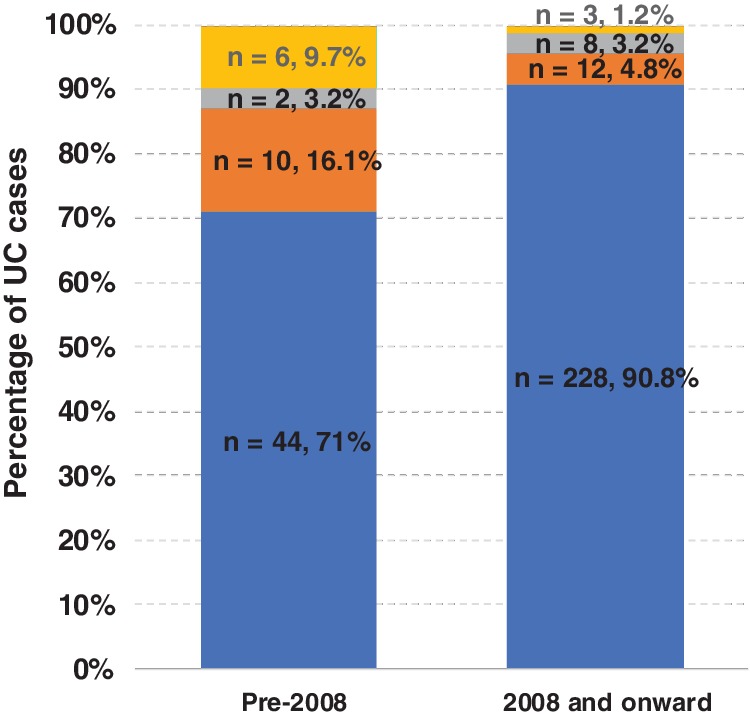
Admission requiring/not requiring surgery pre‐ and post‐2008. 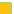, elective; 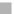, emergent, 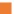, emergency; 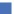, no surgery.

From 2002 to 2007, 19.4% of UC admissions eventuated in emergency/emergent surgery, and this decreased to only 8% from 2008 onward (Table 2). Figure 1 illustrates the reduction in the percentage of cases post‐2008 requiring surgery in comparison to pre‐2008, particularly with emergency and elective surgeries. The rate of surgery throughout the years in the post‐2008 period remained low, except for during 2014–2015 (15%), despite comparable proportions of severe cases (*n* = 45, 62%).

**Table 2:** Primary outcomes

		Emergency/emergent colectomy		
		No	Yes	*P*‐value
Infliximab	Preintroduction	50 (80.6%)	12 (19.4%)	0.008†
	Postintroduction	231 (92.0%)	20 (8.0%)	
Severity of UC	Moderate	96 (96.0%)	4 (4.0%)	0.013†
	Severe	185 (86.9%)	28 (13.1%)	

† Chi‐square test.

UC, ulcerative colitis.

After adjusting for severity and age of patients presenting with UC, the logistic model indicated that there was a 72% reduction in the emergency colectomy rate (OR = 0.28, *P* = 0.005) and a 62% reduction in the rate of emergency/emergent colectomies (OR = 0.38, *P* = 0.017) since the introduction of Infliximab in 2008. Patients with severe UC were 3.5–5 times more likely to undergo emergent or emergency surgery (*P* < 0.05) compared to those with moderate disease. The age at presentation had no significant influence on the likelihood of emergency and/or emergent surgery (*P* > 0.05) (Table 3).

**Table 3:** Logistic regression modeling of emergency and emergency/emergent colectomy pre‐ and postintroduction of infliximab in 2008

		Emergency colectomy only		Emergency and emergent colectomy	
Variable	Category	OR (95% CI)	*P*‐value	OR (95% CI)	*P*‐value
Infliximab	Preintroduction	1.00 (Ref)		1.00 (Ref)	
	Postintroduction	0.28 (0.11, 0.69)	0.005	0.38 (0.17, 0.84)	0.017
Severity of UC	Moderate	1.00 (Ref)		1.00 (Ref)	
	Severe	4.82 (1.09, 21.26)	0.038	3.47 (1.17, 10.24)	0.024
Age at presentation	Range 15–85	1.01 (0.98, 1.04)	0.502	1 (0.98, 1.03)	0.773

1.00 (Ref), reference level; CI, confidence interval; OR, odds ratio.

**Figure 2:**
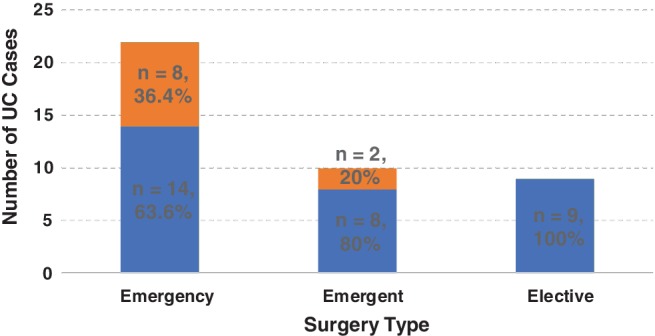
Postcolectomy complications. 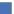, no complication; 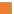, complication.

Only 4 of 159 admissions (0.03%) receiving Infliximab had recorded related complications. Two had a severe drug reaction necessitating immediate cessation, and two had significant infections secondary to immunosuppression. One patient had a peritonsillar abscess and the other oral candidiasis. Ten patients experienced surgical complications, which ranged from superficial wound infections to intra‐abdominal collections (Table 4). Complications were scored using the Clavien‐Dindo classification.^9^ Only two patients who received Infliximab scored >3, while all patients who had complications and did not receive Infliximab scored >3. Overall, there was a 36.4% rate of complications for emergency colectomies compared to 20% for emergent colectomies (Fig. 2). None of the elective operations within these two cohorts had recorded complications. The observed trend in the reduced complication rate was evident. (*P* < 0.095). There was only one mortality, and this occurred in the pre‐2008 cohort following emergency colectomy. The patient was 73 years of age, had significant comorbidities, and presented with a severe flare of UC.

**Table 4:** List of surgical complications

Surgical classification	Surgical complications	TNF‐∝ inhibitor use	Clavien‐Dindo score
Emergent	Small bowel perforation	No	3b
Emergent	Rectal stump leak	Yes	3b
Emergency	Rectal stump leak	No	3b
Emergency	Enterocutaneous fistula	No	4b
Emergency	Anastomotic leak	No	3b
Emergency	Intra‐abdominal collection	No	3b
Emergency	Enterocutaneous fistula	Yes	3b
Emergency	Wound infection (superficial)	Yes	1
Emergency	Wound infection (superficial)	Yes	1
Emergency	Wound infection (superficial)	Yes	1

**Figure 3:**
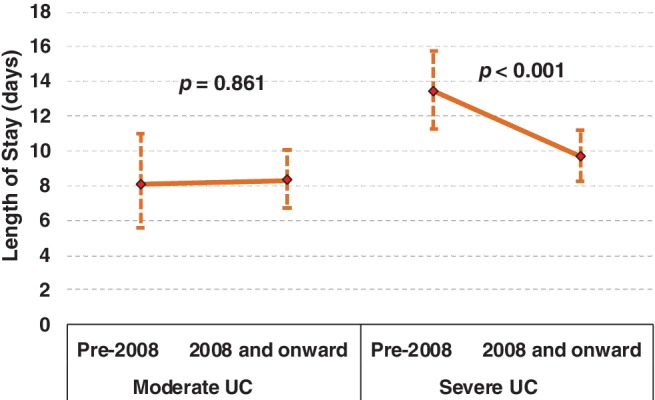
Average length of stay for moderate and severe ulcerative colitis (UC) pre‐ and post‐2008. The error bars represent the 95% CI.

In relation to average length of stay (aLOS), the GLM demonstrated a significant year × severity interaction (*P* < 0.033), where no significant difference in mean aLOS was observed between admissions pre‐2008 (8.1 days; 95% CI 5.6–11 days) and post‐2008 (8.3 days, 95% CI 6.7–10 days) for moderate UC cases; however, a significant reduction in median length of stay (LOS) was evident in severe UC cases post‐2008 (9.7 days, 95% CI 8.3–11.9 days) compared to pre‐2008 (13.4 days, 95% CI 11.3–15.8 days) (see Fig. 3).

Not unexpectedly, the aLOS (16.0 days, 95% CI 13.1–19.3 days) for patients who underwent emergency surgery was significantly higher than those who had emergent (8.9 days, 95% CI 5.8–12.4 days) (*P* = 0.007) or medical management (5.7 days, 95% CI 5.0–6.4 days) (*P* < 0.001). The aLOS is also positively correlated with the age at presentation of UC (*P* < 0.001).

## Discussion

This study confirms the efficacy of Infliximab in reducing emergent and emergency colectomy rates. Prior to the introduction of TNF‐*α* inhibitors, the emergency and emergent colectomy rate at our institution was 19.4%. Our results have shown a significant decline in the rate of emergency and emergent colectomies performed at our institution to 8% post‐2008, which is comparable to other studies.^4^, ^6^, ^10^, ^11^ There have been no prior studies on the efficacy of TNF‐∝ inhibitors in preventing colectomies in Australia.

Although an overall reduction in both emergency and emergent operations was observed, there was also a lower proportion of patients requiring emergency surgery. In the pre‐Infliximab era, the proportion of emergency to emergent operations was 2 to 1. This has changed in the post‐Infliximab era to 1.5 to 1. This meant an increased opportunity for laparoscopic surgery while minimizing LOS, complications, and mortality, which is more achievable in the semielective setting.^12^ There was also a noticeable reduction in the rate of complications from 36% in emergency operations to 20% in emergent operations, and no complications were identified in elective procedures. It is for this reason that we believe the differentiation between emergency and emergent colectomies was important.

It is well established that emergency colectomies in IBD are associated with significantly higher morbidity and mortality compared to the elective setting.^12^, ^13^ Compounding this, the estimated annual hospital cost for the treatment of IBD in Australia is up to $100 million per annum.^14^ As such, this is an important cohort of patients to study.

Although licensed for use in 2008, there was a gradual increase in its use observed over the following years. This is likely due to an increased awareness among prescribers and availability of the drug. In 2013, a specialist referral unit for IBD was established at this institution, which also saw a rise in the number of referrals and admissions. This may partly explain the rise in acute admissions observed in the 2013–2014 period, which can also be attributable to an overall increase in the incidence of UC in Australia.^1^

The introduction of TNF‐*α* inhibitors also resulted in a shorter LOS for patients who were admitted with severe UC. For patients admitted with moderate disease, there was no difference in LOS, which may reflect the fact that only one patient in our cohort with moderate disease was given Infliximab. Furthermore, the average LOS was significantly longer in patients who underwent emergency operations when compared to patients who underwent emergent operations or had no surgery, which is consistent with data published by Patel *et al*.^12^ This has therefore had significant implications on local resources at this institution.

The cost of a 100 mg single dose of Infliximab in Australia is $507.42.^14^ Due to the previously noted high cost of each dose of Infliximab ($507.42), it is a highly regulated drug and is reserved only for severe flares of UC that are resistant to standard medical therapy. However, it is likely that the reduced number of operations, LOS, and complications in our cohort of patients in the post‐Infliximab era will result in tangible savings to the Australian health‐care system, which may balance the cost of TNF‐∝ therapy. Further research is required to confirm the cost‐effectiveness of the use of Infliximab in UC.

### *Limitations*

This study was not without limitations. Being a retrospective study, all data were obtained via medical notes and patient information databases available. The data are therefore limited to the accuracy of the documentation on these platforms. This may have had implications, particularly regarding the accuracy of staging of the severity of acute flares. Our institution is one of three public tertiary referral centers in Western Australia. Patients often attend other institutions in the emergency setting, which may have affected the accuracy of the number of admissions with UC in our catchment area per year. Compounding this, some patients may have eventually had their treatment at private hospitals whose records are beyond the ethical approval of this study.

## Conclusions

Overall, the need for emergency and emergent operations has drastically reduced at our institution with the introduction of TNF‐alpha inhibitors. For a patient who presents with severe colitis, the use of this medication has increased the likelihood that he or she could be managed medically or with an emergent/elective operation rather than an emergency operation with all its associated risks and complications. This study has confirmed the efficacy of TNF‐alpha inhibitors in reducing colectomy rates at our institution. This has elicited a paradigm change in the management of patients presenting with acute severe flares of UC, which were previously associated with significant morbidity. Further cost analysis research would justify its use even more and assess the financial impact of TNF‐alpha inhibitors on the hospital system and patients.
